# Evaluation of Antibacterial and Antibiofilm Activity of Rice Husk Extract against *Staphylococcus aureus*

**DOI:** 10.3390/pathogens13010080

**Published:** 2024-01-16

**Authors:** Gloria Burlacchini, Angela Sandri, Adele Papetti, Ilaria Frosi, Federico Boschi, Maria M. Lleo, Caterina Signoretto

**Affiliations:** 1 Diagnostic and Public Health Department, University of Verona, 37134 Verona, Italy; gloria.burlacchini@univr.it (G.B.); caterina.signoretto@univr.it (C.S.); 2 Nutraceutical and Food Chemical-Toxicological Analysis Laboratory, Department of Drug Sciences, University of Pavia, 27100 Pavia, Italy; adele.papetti@unipv.it (A.P.); ilaria.frosi01@universitadipavia.it (I.F.); 3 Department of Engineering for Innovation Medicine, University of Verona, 37134 Verona, Italy; federico.boschi@univr.it

**Keywords:** *Staphylococcus aureus*, plants waste, biofilm, rice husk extract

## Abstract

Infections caused by *Staphylococcus aureus* are particularly difficult to treat due to the high rate of antibiotic resistance. *S. aureus* also forms biofilms that reduce the effects of antibiotics and disinfectants. Therefore, new therapeutic approaches are increasingly required. In this scenario, plant waste products represent a source of bioactive molecules. In this study, we evaluated the antimicrobial and antibiofilm activity of the rice husk extract (RHE) on *S. aureus* clinical isolates. In a biofilm inhibition assay, high concentrations of RHE counteracted the formation of biofilm by *S. aureus* isolates, both methicillin-resistant (MRSA) and -sensitive (MSSA). The observation of the MRSA biofilm by confocal laser scanning microscopy using live/dead cell viability staining confirmed that the bacterial viability in the RHE-treated biofilm was reduced. However, the extract showed no or little biofilm disaggregation ability. An additive effect was observed when treating *S. aureus* with a combination of RHE and oxacillin/cefoxitin. In *Galleria mellonella* larvae treated with RHE, the extract showed no toxicity even at high concentrations. Our results support that the rice husk has antimicrobial and antibiofilm properties and could potentially be used in the future in topical solutions or on medical devices to prevent biofilm formation.

## 1. Introduction

*Staphylococcus aureus* is one of the most widespread bacterial pathogens, responsible for many types of infections, ranging from uncomplicated skin infections to more severe, invasive infections. E.g., *S. aureus* causes various skin infections, including boils, abscesses, and wound infections, which are usually not life-threatening but can be accompanied by significant morbidity and pain [1,2]. Furthermore, *S. aureus* has been correlated with the development of atopic dermatitis. It is also a major etiological agent in pneumonia and other infections of the respiratory tract, surgical sites, joint prostheses and cardiovascular infections, as well as in nosocomial blood infections [3]. Indeed, infections caused by *S. aureus* still represent one of the main causes of nosocomial infections, in particular bacteremia, especially in subjects with bladder catheters or with skin infections [2]. In this regard, it has been noted that *S. aureus* bacteremia is responsible for more deaths than that caused by the acquired immunodeficiency syndrome (AIDS), tuberculosis and viral hepatitis combined [4,5].

*S. aureus* infections are very difficult to treat due to frequent antibiotic resistance. In particular, methicillin-resistant *S. aureus* (MRSA) represents the most clinically important form. MRSA infections cause increased mortality, morbidity and prolonged hospital stay when compared with infections caused by methicillin-sensitive *S. aureus* (MSSA) [1]. Furthermore, *S. aureus* has the ability to form biofilms, which also favor antimicrobial resistance [6]. The formation of the biofilm involves the production of an exopolysaccharide matrix (EPS) that surrounds the entire structure blocking the access of substances including disinfectants and antibiotics. An aggravating factor is that drugs penetrate biofilm with difficulty, often in sub-inhibitory concentrations, leading to increased biofilm formation and antibiotic resistance, and hampering the course of infection and treatment [7].

It is therefore necessary to develop alternative new therapeutic approaches to counteract the formation of bacterial biofilms and the consequent resistance to antibiotics with alternative substances [8]. In this scenario, medicinal plants can be a rich source of bioactive molecules with potential antimicrobial activity. The use of plant-derived molecules has the advantage of having reduced toxic effects and often activity even against drugs resistant microorganisms. In addition, products of natural origin can also show activity against biofilms formed by several types of bacteria [9].

In the context of natural products, rice husk represents a waste product with good antibacterial and antibiofilm potential. The rice husk is the outer shell of the grain obtained during the first processing phase of parboiled rice, commonly called “husking”. Rice husk contains different polyphenols; in fact, it is rich in phenolic acids, in particular p-coumaric and vanillic acid derivatives [10]. Some polyphenols are known to have antibacterial and antibiofilm activities [11].

In this study, we investigated, for the first time, the antimicrobial and antibiofilm activities of the rice husk extract (RHE) against both MSSA and MRSA clinical strains isolated from skin wounds infections—among the most common infections caused by *S. aureus*—and further evaluated its toxicity in *Galleria mellonella* larvae and interaction with antibiotics in vitro in light of a potential future clinical application.

## 2. Materials and Methods

### 2.1. Extract Sample Preparation

Rice (*Oryza Sativa* L.) husk is the waste resulting from the husking of paddy rice, which is the raw rice after threshing. Using RP-HPLC-DAD-ESI-MSn technology, more polyphenols in the rice husk have been identified, and are mainly hydroxycinnamic acids and flavonoids [12]. The rice husk was provided by an Italian organic farm (Di Cristiana Azienda Agricola, Robbio, Pavia, Italy), dried overnight in oven at 45 °C, minced into powder (particle size lower than 500 µm) and extracted with 80% ethanol (1:35, *w*/*v*), at 90 °C, for 5 min using a Microwave Assisted Extraction system (Ethos LEAN, Sorisole, Bergamo, Italy) [12,13]. The extract was freeze-dried and stored in sterile tubes at room temperature until use. To perform the in vitro tests, RHE was dissolved in 10% ethanol to obtain the following concentrations: 13 mg/mL (20× concentrated than the original concentration of 0.65 mg/mL), 26 mg/mL (40×), 39 mg/mL (60×), 45.5 mg/mL (70×) and 52 mg/mL (80×). Once the preparation was complete, a sterility test on Brain Heart Infusion (BHI) agar medium (Difco Laboratories, Detroit, MI, USA) was performed to verify that the obtained extract was not microbiologically contaminated.

### 2.2. Bacterial Strains

Two *S. aureus* clinical strains, collected from skin infections collected during routine clinical diagnostics at the Microbiology and Virology Unit of the University Hospital of Verona, Italy, were used. Based on their susceptibility to beta-lactam antibiotics, one was classified as MSSA and the other as MRSA. The permission to store and further analyze the isolates is implicitly included in the patient–hospital agreement. Strains used were stored in Microbank (Pro-Lab Diagnostics, Neston, UK) at −80 °C.

### 2.3. Antimicrobial Testing

RHE antimicrobial activity was evaluated using a Kirby Bauer test with a modified diffusion method. Bacterial strains were plated onto BHI agar and grown at 37 °C for 24 h. A single colony was inoculated in BHI medium and grown for 16 h at 37 °C, under shakening. Absorbance at 600 nm was measured, and bacterial cells were diluted to 1 × 10^6^ CFU/mL. A total of 0.1 mL of the bacterial suspension was evenly distributed on BHI agar plates. Subsequently, a drop (10 μL) of each concentration (20×, 40×, 60×, 70× and 80×) of the husk extract was placed on the surface of the BHI agar plates; 10% ethanol (RHE resuspension medium) was used as a negative control. After incubation for 24 h at 37°C in aerobic conditions, the halo zone of inhibition was measured [14].

### 2.4. Growth Curve Kinetics

Growth curves of MSSA and MRSA isolates were measured in presence/absence of RHE. Bacterial cultures were prepared as described above and grown overnight. Absorbance at 600 nm was measured and cultures were diluted to 0.1 OD_600_/mL in 15 mL of a growth medium. RHE 70× was added to the treated cultures; 10% of ethanol (RHE resuspension medium) was used as the negative control. Cultures were incubated for 9 h at 37 °C under stirring (160 rpm) and in aerobic conditions. Every hour, 100 μL of culture were collected, plated on BHI agar and incubated at 37 °C. After 24 h, Colony Forming Units (CFU) were counted. The growth rate was calculated using GraphPad Prism version 7.0 software (La Jolla, CA, USA) [15].

### 2.5. Inhibition of Biofilm Formation

The capacity of RHE to prevent the biofilm formation of MSSA and MRSA was evaluated, as previously described [16]. Briefly, 4 × 10^5^ CFU/mL of bacterial suspensions were inoculated in 200 μL of BHI medium containing different RHE concentrations (60×, 70×, and 80×) in 96-well polystyrene microtiter plates; 10% of ethanol (RHE resuspension medium) was used as a negative control. For each strain, untreated controls were included. Plates were incubated at 37 °C in aerobic conditions for 48 h, with the medium changed every 24 h. After 48 h, the growth medium was removed by aspiration; wells were gently washed with water and air-dried; adherent bacteria were stained with 100 μL of 0.01% crystal violet. After incubation at room temperature for 15 min, wells were washed with water, and 200 μL of ethanol:acetone (8:2, *v*/*v*) were added. Biofilm formation was quantified by measuring the absorbance of the solution at 540 nm. Values obtained in the treated samples were compared with the controls (100%) and expressed as % biofilm inhibition (BI). Experiments were run in triplicate and performed twice. Cut-off values were estimated [17] and used to classify the isolates (untreated) as strong, moderate, low or non-biofilm producers.

### 2.6. Biofilm Disaggregation Assay

Mature biofilms of MSSA and MRSA strains were grown in 96-well polystyrene microtiter plates as described above for 24 h, then RHE was added at different concentrations (60×, 70× and 80×); 10% ethanol (RHE resuspension medium) was used as a negative control. After overnight incubation at 37 °C, the culture medium was removed, and the biofilm formation was quantified as described above. The values obtained in the treated samples were compared with the controls (100%) and expressed as % biofilm disaggregation (BD). The experiments were run in triplicate and performed twice [16].

### 2.7. Confocal Laser Scanning Microscopy (CLSM)

MRSA biofilms with or without RHE 70× were grown as described above, on round plastic slides (20 mm in diameter) using a 6-well microtiter plate as support. After 24–48 h, biofilms were observed by CLSM [18]. The FilmTracerᵀᴹ LIVE/DEAD^®^ Biofilm Viability Kit (Thermo Fisher Scientific, Waltham, MA, USA) was used for biofilm visualization. This kit uses a mixture of two fluorescent dyes: SYTO9 (green fluorescent nucleic acid dye) and Propidium iodide (PI, red fluorescent nucleic acid dye). SYTO9 binds indiscriminately to the entire bacterial population, while PI only penetrates bacteria with damaged cell membrane, causing a reduction in SYTO9 fluorescence. The bacteria with the intact cell membrane (viable) will appear colored in green, while bacteria with the damaged cell membrane (dead) will appear orange-red.

The two fluorescent dyes were added to the RHE-treated and untreated biofilms, and left to incubate at room temperature for 20/30 min in the dark. After gentle washing of the slide with sterilized filtered water to remove excess dye, the slide was observed by CLSM. Z-stack images (1024 × 1024, 10 layers, 6.13 µm distance) were acquired with a LEICA TCS-SP5 Upright Confocal-Multiphoton Microscope (Leica, Wetzlar, Germany). The fluorescent dyes were excited at 488 nm (first channel) and 543 nm (second channel) and visualized with a 20× objective in the range of 500–550 nm (first channel) and 550–620 nm (second channel). LAS X (Leica) version 3.0.16120.2 (Leica Microsystems, Wetzlar, Germany) and ImageJ software version 1.54h (National Institutes of Health, Bethesda, MD, USA) were used to process the image data.

### 2.8. Toxicity in Galleria mellonella Larvae

Ten larvae were inoculated with different concentrations of RHE (60×, 70× and 80×), through the last proleg into the hemocoel by using a 0.3 mL syringe, and incubated on filter paper in Petri dishes, at 37 °C, in the darkness. As control groups, ten larvae were inoculated with sterile saline solution, and ten with 10% ethanol (RHE resuspension medium). The larvae were then monitored daily for up to 72 h to assess their viability, and death was assessed based on a lack of movement and blackening [19].

### 2.9. Checkerboard Test

The Checkerboard testing method was used to evaluate synergism between RHE and three antibiotics for which resistance against MRSA was observed. For this purpose, two beta-lactam antibiotics, oxacillin (MIC 4 mg/mL) and cefoxitin (MIC 16 mg/mL), and quinolone antibiotic ciprofloxacin (MIC 128 mg/mL), were used. A total of 0.5 McFarland bacterial cells were cultured in 200 μL BHI in a 96-well plate for 16 h at 37 °C with decreasing concentrations (starting from the MIC) of RHE and Oxacillin/Ciprofloxacin/Cefoxitin along the ordinate and the abscissa, respectively. The MIC was defined as the lowest concentration of antibiotic that completely inhibited the growth of the organism as detected with the naked eye. The Fractional Inhibitory Concentration (FIC) Index (FICI) was calculated to evaluate synergy as follows: the combination is considered synergistic when the FICI is ≤0.5, additive when the FICI is >0.5 to ≤1, indifferent when the FICI is >1 to ≤ 4, and antagonistic when the FICI is >4 [20].

### 2.10. Statistical Analysis

Statistical analysis was performed using GraphPad Prism version 7.0 software (La Jolla, CA, USA). Bacterial growth rate was analyzed by one-tailed *t*-test. *p* values < 0.05 were considered to be significant.

## 3. Results

### 3.1. Antimicrobial Activity

The evaluation of the antimicrobial activity of RHE at different concentrations (20×, 40×, 60×, 70× and 80×) against the MRSA and MSSA strains showed that RHE is able to inhibit their growth at high concentrations, which are 60, 70 and 80 times more concentrated than the concentration of the extraction usually present in nature (60×, 70× and 80×) (Table 1). These concentrations, considered to be the most promising, were selected for use to test RHE antibiofilm activity and toxicity.

**Figure 1:**
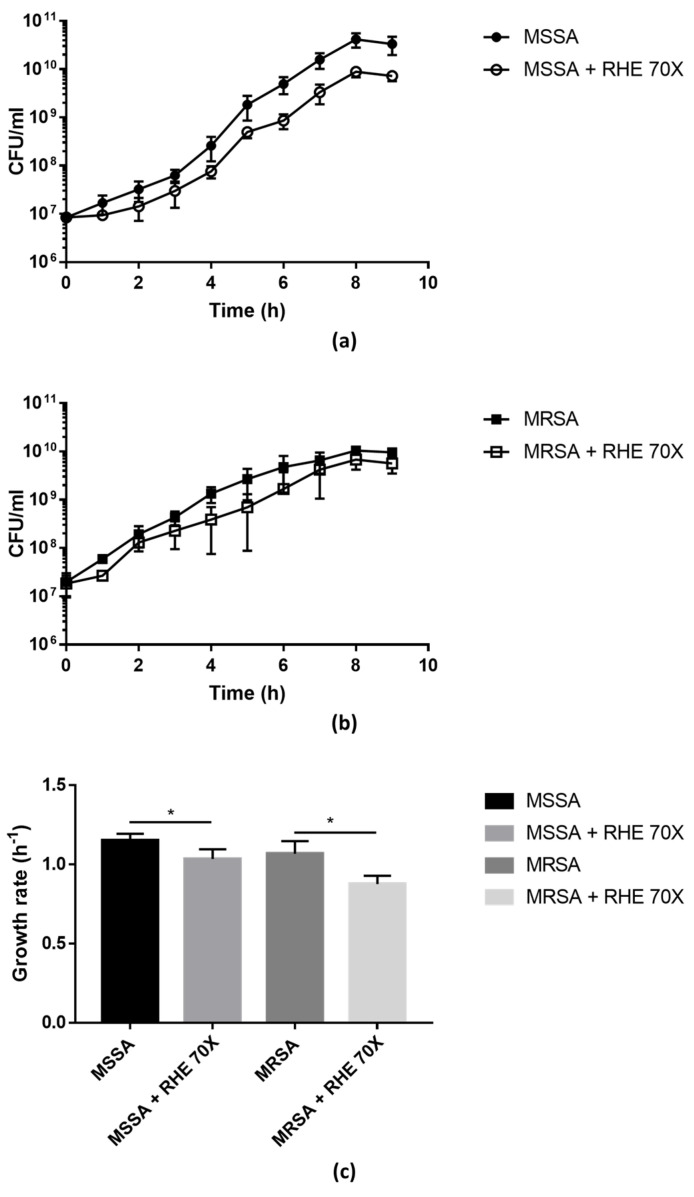
Growth curves of MSSA (**a**) and MRSA (**b**) isolates grown in absence/presence of RHE 70×. Colony-forming units (CFU) were counted after plating serial dilutions collected from the cultures every hour for the first 9 h. Growth rate (**c**) was calculated from the exponential phase of the growth curves. Each value represents the mean ± SD of three experiments. Growth rate in absence vs. presence of RHE 70× was analyzed by one-tailed *t*-test; * *p* < 0.05.

The growth curves of MSSA and MRSA isolates were evaluated in the presence/absence of RHE 70× (sub-MIC concentration). As shown in Figure 1, the presence of RHE significantly affected the bacterial growth compared to the untreated cultures, confirming that RHE has bactericidal effects against *S. aureus*.

### 3.2. Antibiofilm Activity

The clinical isolates used in the study were classified as strong biofilm producers based on the criteria proposed by Stepanovic and colleagues [17]. The antibiofilm activity of RHE at different concentrations was evaluated by both the inhibition of biofilm formation and the disaggregation of pre-existing biofilm. As shown in Table 2, RHE showed inhibition of biofilm formation of both MSSA and MRSA isolates. The inhibition increased with the increase in RHE concentration, going from 5 to 8% inhibition at 40×, 25–30% at 60× and 65–75% at 80×. As shown in Table 3, however, RHE shows reduced activity in destroying pre-existing biofilm in both *S. aureus* strains (only 20% disaggregation at 80×).

The biofilm formed by the MRSA isolate was treated or not with RHE 70× (sub-MIC concentration) As shown in Figure 1, the presence of RHE significantly affected bacterial growth compared to the untreated cultures, confirming that RHE has bactericidal effects against *S. aureus*.

**Figure 2:**
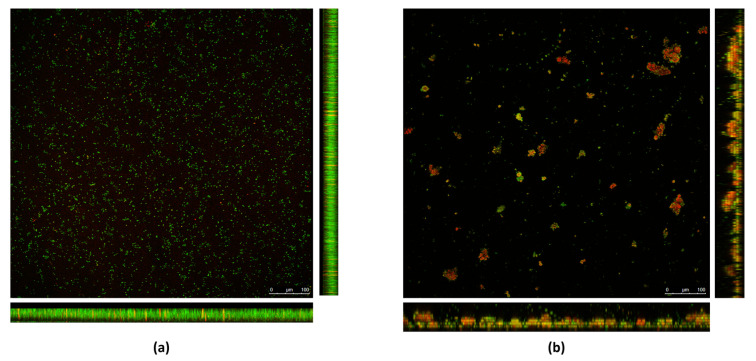
Biofilms formed by the MRSA isolate in (**a**) absence and (**b**) presence of RHE 70×. Bacterial cells are stained with SYTO9 green fluorescent dye. Damaged cells are counter-stained with propidium iodide (PI) red fluorescent nucleic acid dye.

The 70× RHE was analyzed by CLSM; substantial differences were observed between the RHE-treated and the untreated biofilm. As shown in Figure 2a, in the untreated biofilm, bacterial cells formed a homogeneous layer, and were mostly green in color, indicating that the bacterial cells are intact and therefore viable. As shown in Figure 2b, when treated with RHE 70×, a loss of homogeneity in the biofilm layer was observed. Furthermore, aggregates of red-colored bacterial cells formed, meaning that the cells were not intact and therefore presumably dead.

### 3.3. Toxicity

Toxicity of RHE at high concentrations (60×, 70× and 80×) was evaluated in the *G. mellonella* larvae model. RHE induced no toxicity, even at the highest concentration tested. As shown in Table 4, all larvae treated with 60× and 70× concentrations survived up to 72 h. Only 1 out of 10 larvae treated with RHE 80× died at 72 h; the same happened in the control group treated with 10% ethyl alcohol (RHE suspension medium).

### 3.4. Interaction with Antibiotics

The possible synergism of RHE with antibiotics oxacillin, ciprofloxacin and cefoxitin was evaluated on the MRSA strain using the Checkerboard test. The combination of RHE 80× with beta-lactam antibiotics oxacillin and cefoxitin produced an additive effect (FICI = 0.875 and 1, respectively), while the combination with quinolone antibiotic ciprofloxacin had an indifferent effect (FICI = 1.75).

## 4. Discussion

The evolution and spread of the phenomenon of antibiotic resistance constitutes a threat to public health on a global scale, as it reduces the effectiveness of treatments, increasing the mortality and morbidity of infections and the costs of health care [21].

This is compounded by the fact that many bacteria form biofilms which make them even more resistant and refractory to antibiotic treatment. It is known that more than 80% of bacterial infections are mediated by biofilm [22]. It is therefore necessary to develop new strategies in order to prevent and treat microbial infections.

Identifying new compounds able to contrast the biofilm formation could be useful in the prophylaxis to avoid skin or implants of biofilm-associated infections caused by *S. aureus* contamination [22]. Bioactive compounds from plant tissue have been reported to exhibit antimicrobial activity [23]; therefore, they could represent potential new effective and safe, natural antimicrobial compounds. Several studies have reported the antimicrobial activity of fruit and vegetable by-products such as hazelnut peel, pomegranate peel, apple peel, potato peel, leek leaves, dogwood seed, rosehip seed, pomace, rosehip pulp, dogwood pulp, pomegranate pulp, apple pulp and potato pulp [24]. Also, vegetable waste including trimmings, peels, stems, seeds, shells, bran and residues left over after extraction of juice, oil, starch, and sugar have also been shown to be an excellent source of bioactive molecules [25]. The biomolecules of the recovered by-products can be used to produce functional foods or medicinal and pharmaceutical products [26].

In recent years, the interest of the scientific community towards plant compounds and their antimicrobial activity has indeed increased. This may be due to the perception that natural products are healthy and safe for humans and have been used for centuries in traditional medicine of various popular cultures. Recent studies reported that many plant extracts (*Moringa oleifera* leaves, *Vernonia amygdalina*, *Azadirachta indica* and *Acalypha wilkesiana*) possess antibacterial activity against multidrug-resistant *S. aureus* (MDR), in particular against strains associated with skin and soft tissue infections [27,28]. Moreover, the aqueous extracts obtained from soybean residues have shown the ability to effectively prevent the formation of *S. epidermidis* and *S. aureus* biofilms [29].

In a preliminary screening of plant-based bioproducts with potential antimicrobial or antibiofilm activity, we tested the antimicrobial and antibiofilm activity of different plant wastes such as rice husk extract (RHE), melon peel and wheat grain peel against *S. aureus* clinical strains isolated from skin wound infections (Among the different plant compounds tested, we observed promising results only for RHE and therefore, we proceeded to study the antimicrobial and antibiofilm activity of RHE against both MSSA and MRSA, to investigate a potential future clinical application. The antibacterial potential of rice husk is also further supported by a recent study where rice husk liquid smoke has been shown to inhibit the growth of *S. aureus*, *Bacillus subtilis*, *Escherichia coli*, and *Salmonella* spp. [30]. Rice husk is a multi-layered casing, tending to brown or yellow, which covers the grain of rice and constitutes 20% of the total weight. It is removed from the grain of rice during the first stage of processing, called “rice-milling”. It contains lignin, cellulose, hemicellulose, and also a high numberof polyphenols, mainly hydroxycinnamic acid derivatives, which can be free or bound to the polysaccharide skeleton [31,32]. In literature, antibacterial activity against Gram-positive bacteria has been reported and attributed to these acids [33].

In this study, we evaluated the antibacterial and antibiofilm activity of RHE on *S. aureus* strains isolated from skin wounds. “In vitro”, RHE elicited antibacterial activity at high concentrations (70–80×, corresponding to 45.5–52 mg/mL) against both *S. aureus* strains used (MSSA and MRSA). Indeed, the presence of RHE significantly affected bacterial growth compared to untreated cultures, confirming that RHE at high concentration has bactericidal activity against *S. aureus*. Moreover, it showed the ability to inhibit the biofilm formation of both *S. aureus* isolates. CLSM imaging of biofilms with “live and dead” staining further highlighted that RHE interferes with MRSA biofilm formation. In fact, in the MRSA biofilm treated with RHE, a loss of homogeneity in the biofilm layer was observed, together with the presence of clusters of dead bacterial cells. Therefore, our results support the idea that RHE could be used in clinical practice to contrast infections caused by biofilm-producing microorganisms like *S. aureus*, even when antibiotic resistance is observed. In contrast, no or little biofilm disaggregation activity of RHE against these bacterial strains was observed. This discrepancy confirms the knowledge already acquired through previous studies, i.e., the greater difficulty in disrupting an already formed and mature biofilm rather than interfering with its formation phases [34]. This argues in favor of the possible use of RHE to prevent the formation of biofilms on biomedical devices. Furthermore, our “in vivo” data indicate that RHE is not toxic at the concentrations used in the study. Indeed, in *G. mellonella* larvae model there was no difference in survival between RHE-treated and untreated larvae (Table 4).

In addition, we observed promising preliminary results from the combination of RHE with antibiotics against MRSA. The combination of RHE with the beta-lactam antibiotics, oxacillin and cefoxitin, produced an additive effect (FICI = 0.875 and 1, respectively). Therefore, beta-lactams in particular, could be further explored for the development of new therapeutic options, as this approach could be used for the reuse of old drug molecules.

Overall, our results support that RHE is endowed of antimicrobial and antibiofilm activity against *S. aureus*, similarly to many other natural products of plant origin that are known to possess antimicrobial properties and/or anti-biofilm properties in vitro. The antibiofilm effects of natural products are mainly based on the inhibition of the polymer formation matrix and suppression of cell adhesion and attachment, by interrupting the generation of extracellular matrix and decreasing the virulence factor production, thus blocking the quorum sensing network biofilm development [35].

Although the mechanism of action of RHE has not been clarified yet, we speculate that this natural compound could be used in the future for preventive purposes such as in topical solutions or to be applied on medical devices in order to prevent the formation of bacterial biofilm. Further studies are encouraged, using more strains and including different microorganisms, to fully understand the potential of RHE as an antimicrobial and antibiofilm agent.

## Figures and Tables

**Table 1:** Antimicrobial activity of RHE at increasing concentrations. The measure of the inhibition halo (mm) for MSSA and MRSA isolates is shown per each concentration.

RHE Concentration	MSSA	MRSA
20×	0	0
40×	4	3
60×	10	9
70×	13	12
80×	15	14

**Table 2:** Inhibition of biofilm formation at increasing concentrations of RHE. The percentage (%) of biofilm inhibition for MSSA and MRSA isolates is shown per each concentration. Reported data are the mean values obtained from triplicate experiments performed twice (mean ± SD).

RHE Concentration	MSSA (%)	MRSA (%)
20×	0 ± 0.2	0 ± 0.5
40×	5 ± 1.2	8 ± 1.0
60×	25 ± 4.2	30 ± 2.2
70×	45 ± 3.7	50 ± 3.2
80×	70 ± 5.9	75 ± 3.8

**Table 3:** Disaggregation of biofilm at increasing concentrations of RHE. The percentage (%) of biofilm disaggregation for MSSA and MRSA isolates is shown per each concentration. Reported data are the mean values obtained from triplicate experiments performed twice (mean ± SD).

RHE Concentration	MSSA (%)	MRSA (%)
20×	0 ± 0.3	0 ± 0.4
40×	3 ± 0.7	3 ± 0.9
60×	9 ± 1,0	8 ± 1.1
70×	15 ± 1.7	12 ± 2.0
80×	20 ± 1.8	20 ± 1.5

**Table 4:** Toxicity of increasing concentrations of RHE in *G. mellonella* larvae. The number of dead larvae at each time point (24, 48 and 72 h) is shown per each RHE concentration (60×, 70× and 80×) and control group.

Treatment	24 h	48 h	72 h
Sterile saline solution	0/10	0/10	0/10
Ethanol	0/10	0/10	1/10
RHE 60×	0/10	0/10	0/10
RHE 70×	0/10	0/10	0/10
RHE 80×	0/10	0/10	1/10

## Data Availability

Data are available upon reasonable request to the corresponding author.
